# Supplementary material for: Patterns of Intron Gain and Loss in Fungi
Source: PLoS Biol. 2004 Nov 30;2(12):e422. doi: 10.1371/journal.pbio.0020422 (PMC532390; doi:10.1371/journal.pbio.0020422)
Supplement: Table S1 — Also available at http://genes.mit.edu/NielsenEtAl/. (4.3 MB ZIP). [file pbio.0020422.st001.zip › NielsenEtAl/html/1434.html]

AN6675.1.NCU00045.1.MG10196.1.FG07232.1


```
 CLUSTAL W (1.82) Multiple Sequence Alignments - Introns Inserted


Sequence 1: NCU00045.1	1261 aa
Sequence 2: MG10196.1	1217 aa
Sequence 3: FG07232.1	1170 aa
Sequence 4: AN6675.1	1173 aa
Alignment Length: 1318 aa
Number Identitical Residues: 382 aa
Alignment Score (without introns) 21537


MG10196.1 	MPSAPSTPQRPTPARRRGRPRTSTSAARS----SRVAVADTEPPPKKRRYIP-------G
NCU00045.1	MAPVPSTPRRRPQSRPKARSRAGGSATTSRGKRHASGAPATEPPLKKRRYVP-------G
FG07232.1 	MAPASPTPRRPVTGRRRGRPPGSTNAARAARAAALAAASATEPPPKRRRYAPAGSRFIAG
AN6675.1  	MAPYTSPKRSSPGGPLKNGSKTPLLDSGNS---ETPVADNLGPIIKKRKYVP-------G
          	*.. ... :    .  :  .      :          .    *  *:*:* *       *

MG10196.1 	GPGGGGRFVDENGNEVPR------------------PAGSSSAPRPRAAAGTPSTGKPRR
NCU00045.1	GPGGGGRFIDEDGVETPAE-----------------PSGSASRIRTSAARASPS------
FG07232.1 	GAGGGGRYVTSDILATPN------------------TAGPSTSSRSRAAAREAINGP---
AN6675.1  	GPGGGGRYIEIEVRETPKQPKPKPKPKQPKPSPVKRTSSSISRTRHQLREAESSAQPAQL
          	*.*****::  :   .* .............:. . .:.. :  *       .    .  

MG10196.1 	SLAAGSTAVYPRRERSTRMRTATSRFEPDDEQFSSAAAITSAVIQSEGYKPREERGWEEF
NCU00045.1	--------VFPKRERSTRIRTAVNRDKLDDMQYSSAAAVVAAVVQSEGYKPREERGWEEF
FG07232.1 	-----SPSLMPRRERGARTRAAGNE-DLEEMQWGSAAAMATAVKQAEDYKPREERSWEDF
AN6675.1  	P-PPPPPVHAPTTPPSTRLRREKSQ---TRGRFGSSTAAALALQQGDGYKPREERGWEEF
          	. .. ..   *    .:* *   ..      ::.*::* . *: *.:.*******.**:*

MG10196.1 	HPNLDIEATFMVFQSEDVDGTARPRPSTPKYAVPAASADTSTPKG--ASALKTQEDEEAT
NCU00045.1	HPNLDIEGTFMIFQADEVDGILREAPHTPAVQQQPQQHQLGGQDHGATTPTKSAINNMVS
FG07232.1 	HPNLDIEATFLVLRSEQVDGIPQEQPDMSTVPMLTTPLDD-------TRTPSRQPNPAST
AN6675.1  	HPELDIDAQFPVFPSAEVD----RPPLSAQLASILSP---------------NGLDKTNE
          	**:***:. * :: : :**      *  .                          :    

MG10196.1 	KDAALVPPEGSQN------STTTPMTRRSRGRPSRDPLGVYGMRGADS-TTPKVPKVLPI
NCU00045.1	TDAPNAATPSGQDRPLLAGSVTDTPSRRRPLRPTRESSSIYANRPLDFGITPKTPKVLPI
FG07232.1 	GNTPNPQGRSDSN----AADAPNETPLRRPRRPTRDVVSFYSSRPLDLMTTPKTPKILPI
AN6675.1  	NDPVAELIRAHAN------GTVQTPIKRRPGRPPRRPEAILQS----LGVFTPQPKVVPP
          	 :.      .  :      ..      *   **.*   ..           .  **::* 

MG10196.1 	HNQTPKERLDLKQPSYRKTDRISLFESKTFGQARYVEKSMMNVGYQESDQYLRPERMLIK
NCU00045.1	HNQTPKERLDLKQPSYRKTDRIALFESKTFGQARYVDKSMSNVGYQESDNFIRPDRKLIK
FG07232.1 	QNQTPKEKLDLKLPSYRKTNRIELFESKTFGQARYVDKAMSNVGYQESDHYMRPDQSLIK
AN6675.1  	PGPNPREKLTLPKPSFRLKDPFTFYDQPGVGQQNYVDRTMASVGYQESDLFIRHDRRLIR
          	 . .*:*:* *  **:* .: : :::.  .** .**:::* .******* ::* :: **:

MG10196.1 	ATDGNIEEDLEQLSAIAKVDGATTSPASNALGRVEYDMDEQDDMWLEKYNERRKEASLDT
NCU00045.1	ATDANVEEDIDP-STMTQANSDSAHNSAGAVGRVEYDMDEQDDMWLETLNRRRKDSNLDT
FG07232.1 	SSDLHAEDDADLTDATASEEPVSHR----RIGRVEYDMDEQDDMWLERLNVRRKEDELEE
AN6675.1  	MPEGAQEDDLDIGNPLLGEGEVNAA-----VGRVEYDMDEQDEKWLEDYNAKRREDQYEP
          	 .:   *:* :  ..       .       :***********: ***  * :*:: . : 

MG10196.1 	ITREIFEIAITRIEKEWHALEKRIPKPNPKPPQTHRPRSSSAAAVNGETQA-GEEQDSKC
NCU00045.1	ITREIFEITITKIEKEWHALEKRIPKPNPKPPQTHRPRSSSAAAVNGEPQT-GEEQDSKC
FG07232.1 	ITREIFEITMTKIEKEWHALEKRIPKPNPKPPQTHRPRSSSAAAVNGEPQG-GEEPDSRC
AN6675.1  	IKPAVFEITMTKIEKEWHSLEKRIPKPNPKPPQTQRPRSSSAAAVNGETTGPGEDQDTKC
          	*.  :***::*:******:***************:*************.  .**: *::*

MG10196.1 	AICDDGDCENTNAIVFCDGCDLAVHQECYGVPFIPEGQWLCRKCQLIGRGVP0TCIFCPN
NCU00045.1	AICDDGDCENTNAIVFCDGCDLAVHQECYGVPFIPEGQWLCRKCQLIGRGVP~TCIFCPN
FG07232.1 	AICDDGDCENTNAIVFCDGCNLAVHQECYGVPFIPEGQWLCRKCMLCGRGVP0TCIFCPN
AN6675.1  	AICDDGDCENANAIVFCDGCDLAVHQECYGVPFIPEGQWLCRKCQLIGRGSP~NCIFCPN
          	**********:*********:*********************** * *** * .******

MG10196.1 	TDGAFKQTNSSKWAHLLCSMWIPEVSLGNHTFMEPVMDVEKVPKTRWKLTCYLCNQRMGA
NCU00045.1	TDGAFKQTNSSKWAHLLCAMWIPEVSLGNHTFMEPVMEVEKVPKNRWKLVCYICNQKMGA
FG07232.1 	TDGAFKQTNSSKWSHLLCAMWIPEVSLGNHTFMEPVMDVEKVPKSRWKLTCYICRQRMGA
AN6675.1  	IEGAFKQTTTSKWSHLLCAIWIPEVSIGNPSLMEPITDVEKVPRSRWKLQCYICRQKMGA
          	 :******.:***:****::******:** ::***: :*****:.**** **:*.*:***

MG10196.1 	CIQCGNKACYQAFHVTCGRRARLYLKMKNSQGALAVLDGNMVLKAFCDKHCPADYTKENN
NCU00045.1	CIQCSNKNCFQAFHVTCARRSRLYLKMKNSQGALAVLEGGLPLKAYCDKHCPQDYAKEND
FG07232.1 	CIQCGNKNCYQAFHVTCARRSRLFLKMKTSQGALAVLDGGMVLKAFCDKHCPPDYAQEHN
AN6675.1  	SIQCSNKNCFVAFHVTCARRAQLYLKMKSGHGNLAVMDS-HLLKAFCDKHVPPEWRREHG
          	.***.** *: ******.**::*:****..:* ***::.   ***:**** * :: :*:.

MG10196.1 	VAQATRDAKKFYKKMMKGRLWATSQDSAAELAATHRQALHDQPADDLQATGDN-SVAAAD
NCU00045.1	VVQATKDAKRFYKRAMKGRIWADSQASALQLAATHRHAITEHPPDESQMTGAKVSAVLGE
FG07232.1 	IHQATKAAKKFYKRTMRNRIWADNTVAANNIAAQHRDALAEQPSEETQLAGNK-SSISGD
AN6675.1  	TDAATADAIEYYRTTMQGRRWGDSQAAALSLEPAHPLGGGPEDEDGQRTHTPRITLTVGG
          	   **  * .:*:  *:.* *. .  :* .: . *  .   .  :  :    . :   . 

MG10196.1 	KKRGQV--QKCWWRLPSGAPVIPQSVLTIVEGAMQRFPFSKRKDFVAEACRYWTLKREAR
NCU00045.1	TKNGHP--GKPIWKLPSGAPIIPQAVYDLVESALARFPILKRKDFVAEACRYWTLKREAR
FG07232.1 	KKKGQP--PKNLWKMPSGAPVIPQVVFEIVEASIQRFPFRKRKDFLSEACRYWTLKRQKR
AN6675.1  	NKRKRPNAPRTIWKLPSGAPVIPQVLLNSVVASLQRFGVRQRKQYAEDACKYWTLKREAR
          	.*. : .: :  *::*****:*** :   * .:: ** . :**::  :**:******: *

MG10196.1 	RGAALLKRLQLQMETFSSMELTRRDFSAMGQSGKARLERRIEFANMLIKDLEQLKTLSDE
NCU00045.1	RGAALLKRLQLQMETFSSMELTRRNFAAMGPSGKTRLARRIEFARTLLKDLEHLKALSEE
FG07232.1 	RGAALLKRLQLQMESFSSMELTRRDFAAMGPSGKARLTRRIEFAEDLVKELEQLRNLASQ
AN6675.1  	RGAALLKRLQLQLETFSSMEMTRRDYAAMGATGSKRLQRRIEFGDRLYRDLDRLRMLCDE
          	************:*:*****:***:::*** :*. ** *****.  * ::*::*: *..:

MG10196.1 	IVKRELDKLEAAELEQDFVDTVYFPIYKLLLPVIDKAFA2LD-KSVFQEGLNGLQNRLDE
NCU00045.1	IVQREASKLEAAEMERDFVDSCYFPVYEMLIPALQKAIQ2SD-RDVFAKGLSELQDKMEK
FG07232.1 	VVEREQIKVDAGELEQEFVDECYFPVAKLLDPAIDRAIS2LD-KDLFSNGLDKLQTRINT
AN6675.1  	VKKREREKLKDAEMLRNIVDTVYFPIFPLLWPIFEKAQV2LDSKGFFRRGLLAIRTKLEQ
          	: :**  *:. .*: :::**  ***:  :* * :::*    *::..* .**  :: ::: 

MG10196.1 	RHYTTTLAFAHDLCEVIHVGINSSTIKPDVVELSDRPEEGEALDVSPSKQGSGYVEARDR
NCU00045.1	RYYTTTLQFTHDLCQVISAEINSAMNPHAGLDQIVEPS-----YASPTKPGTN-AEVKKC
FG07232.1 	RFYVTVMSFAVDLCKVISDGVATTPEPKLSTDATQHESN----DASPAKHTFS--DIRER
AN6675.1  	RYYPSVSSFSAHLASIFTSEIGVQPAG-DTAELQMQISG--------RAPELS-LEQREK
          	*.* :.  *: .*..::   :          :   . .              .  : :. 

MG10196.1 	KRLGKRILKAVQPQLETALHAEADIANKPFEKLVKELEGMLEASLELRQASITVSQDENV
NCU00045.1	KQMGKRILRQVQPYLEAALQAEADICSKPYDTLHQELEGMLDASLEVRQPTITVSSHDGV
FG07232.1 	RKLGKRILKAVQPFLEAALRVESEISQKPFEGLQKELEDTIDKSVESRRPLTATSQDKST
AN6675.1  	RKLAKRIIKAIQPALEDAIRKESELTRQPFEQELKDLDVMLGDVSSGRGSEIDSAERPGE
          	:::.***:: :** ** *:: *:::  :*::   ::*:  :    . * .    :.  . 

MG10196.1 	APEHNGEVAMVDAPDVDAGQIIVADH-------------ETRTTHHVDAQADVDRMQIDV
NCU00045.1	AAED---VEMADAPE-EGGQIIVADESDGEADGDGDEDGDGKADANVDSNGEPDEMNVDT
FG07232.1 	DPSD------------EANDTIMVDA-------------ELQITVKADSTEGGDAMDITS
AN6675.1  	EAFE--------KSELPNGDTDSTNM-------------DPNSTAKMKSTEEVLESTETN
          	 . .         ..   .:   .:              : .   : .:           

MG10196.1 	AVDDAGN--IEVDTSGVENAVVEVNGCASSTSSVAGQE-NQPTDLTKLDVHLTNGTPKQS
NCU00045.1	SQQKAGVGSIEVNTSEVD-AAPESNNTLSTSQSVAGEEEKENHKEDSQQLQLPDGPTKDS
FG07232.1 	---DDGN--IDVST-----------NIDVDTSEIAKAEAGEKQE------SLPN-TVQSS
AN6675.1  	---TPGAEPSKLTGS----QAAQAAATENTPVDVPMSDASTTQPPNEAQAAITPELAEEP
          	     *  . .:  :       .       . .:.  :        . .  :.    :..

MG10196.1 	GTPP-----ADSNYMHPQLPSGPQPGPLTPPQSNGGLGAGGASGPADVLTDGGLPWYLKS
NCU00045.1	GTPPSLPGEGPDRANVPQLSHPQQSGPLTPPQSNGSFGR---DPTTNILTEGGVPTYLHG
FG07232.1 	DTPP-----GTDGYVS--KPQTAQSGPPTPPQSNGSLGLD----PSDPLTDGGILWYLKG
AN6675.1  	GEAP----------VIQQQTNQTPQVPLSPPALET--------DQHHPLARGGIQWYMQP
          	. .*             . .      * :**  :            . *: **:  *:: 

MG10196.1 	FHVDGTSAAEDKWAGRDAVRSLSEDLTDMDEEELNGLEFDVEDS-TITASPVN-------
NCU00045.1	FQIEGTSVIEEQWPGREAVRSLSEELTDMDDEALKDLEFDVDEADTITATSPNHVPVPLG
FG07232.1 	LDPKGTSVLEEQWAGRDAVRTLSEDLTDLDDEALKGLGMDVDNAVASAAVEAD-------
AN6675.1  	FDPMGTTIHEERWTGREVMRGLSEELSELDEEELNDLVDEDELEENATGTGVF-------
          	:.  **:  *::*.**:.:* ***:*:::*:* *:.*  : :     :.           

MG10196.1 	-----------------------------ASSSGPMLSTRKRSISSVRHRKGVRTSARRR
NCU00045.1	TAEIAVEGGASSSKDVAAAVVTAAVANATATLSAPPVTRRRERANPAMARKGVRSSARRR
FG07232.1 	-----------------------------EKEEVKAAAEPVGGKTRASKAKKRRASTRRR
AN6675.1  	------------------------------ANRAPDSSRGEQGVKVHRTRRRWRGFK---
          	                                     :      .     :  *      

NCU00045.1	
MG10196.1 	
FG07232.1 	
AN6675.1  	
          	
```
